# Tailored antisense oligonucleotides designed to correct aberrant splicing reveal actionable groups of mutations for rare genetic disorders

**DOI:** 10.1038/s12276-024-01292-1

**Published:** 2024-08-01

**Authors:** Htoo A. Wai, Eliska Svobodova, Natalia Romero Herrera, Andrew G. L. Douglas, John W. Holloway, Francisco E. Baralle, Marco Baralle, Diana Baralle

**Affiliations:** 1 Human Development and Health, Faculty of Medicine, University of Southampton, Southampton, UK; 2 Department of Experimental Biology, Faculty of Science, Masaryk University, Brno, Czech Republic; 3 Department of Clinical Immunology and Allergology, Faculty of Medicine, Masaryk University, Brno, Czech Republic; 4 Oxford Centre for Genomic Medicine, Oxford University Hospitals NHS Foundation Trust, Oxford, UK; 5 Fondazione Fegato, Area Science Park Basovizza, 34149 Trieste, Italy; 6 International Centre for Genetic Engineering and Biotechnology (ICGEB), Padriciano 99, 34149 Trieste, Italy

**Keywords:** Drug development, Antisense oligonucleotide therapy

## Abstract

Effective translation of rare disease diagnosis knowledge into therapeutic applications is achievable within a reasonable timeframe; where mutations are amenable to current antisense oligonucleotide technology. In our study, we identified five distinct types of abnormal splice-causing mutations in patients with rare genetic disorders and developed a tailored antisense oligonucleotide for each mutation type using phosphorodiamidate morpholino oligomers with or without octa-guanidine dendrimers and 2′-O-methoxyethyl phosphorothioate. We observed variations in treatment effects and efficiencies, influenced by both the chosen chemistry and the specific nature of the aberrant splicing patterns targeted for correction. Our study demonstrated the successful correction of all five different types of aberrant splicing. Our findings reveal that effective correction of aberrant splicing can depend on altering the chemical composition of oligonucleotides and suggest a fast, efficient, and feasible approach for developing personalized therapeutic interventions for genetic disorders within short time frames.

## Personalized medicine: targeted oligonucleotides for rare genetic disorders

Millions globally suffer from rare diseases, often genetic and affecting children. This study explores using antisense oligonucleotides to fix incorrect RNA splicing, a common result of disease-causing genetic mutations. The results showed that tailored ASOs could correct incorrect splicing for various mutation types, showing this technology′s potential in treating rare genetic diseases. The team chose five mutation types disrupting normal splicing and created specific ASOs to correct these errors in cell models. They created minigenes to simulate the mutations and tested different ASOs′ effectiveness. This method was key to understanding ASOs′ ability to restore normal gene function, crucial for developing targeted treatments for rare genetic disorders. This research could lead to new, targeted treatments for rare genetic disorders, offering hope to millions of patients and their families facing limited treatment options. This summary was initially drafted using artificial intelligence, then revised and fact-checked by the author.

## Introduction

It is estimated that 263 to 446 million people worldwide are affected by rare diseases, with 71.9% having genetic origins and 69.9% of these patients being children^1^. Most rare disease patients harbor unique mutations in their genomes^2^. However, the development of therapeutics for rare diseases lags significantly behind their diagnosis^3^. Only 6% of these diseases have known treatments^4^, and 30% of affected individuals do not survive to see their fifth birthday due to the lack of available treatments^5^. It is therefore imperative to expedite the development of life-saving treatments as soon as rare disease patients are diagnosed.

Although genetic mutations can cause diseases in various ways, many mutations impact RNA splicing^6,7^. RNA splicing is a posttranscriptional process in which intronic sequences are removed from pre-mRNAs and exons are joined together^8^. The splicing process is regulated through sequence recognition by splicing factors^9^. Among the more important sequences for splicing are the 5′ and 3′ splice sites. Mutations falling within these cis-acting elements can lead to aberrant splicing in patients with genetic disorders^10,11^ and are important targets for therapeutic development aimed at correcting aberrant splicing. Alternatively, mutations may also create or disrupt cis-acting elements that alter physiological splicing.

Antisense oligonucleotides (ASOs) were first proposed for their therapeutic potential by directly targeting specific RNAs^12^. ASOs possess both target RNA binding affinity and specificity through Watson–Crick base-pairing^13^. Antisense technology has evolved into a favorable therapy for treating patients with rare and common diseases^14^ and has become the treatment of choice for “n = 1” patients^15^. A chemical property of ASOs is their ability to occupy sequence motif binding sites in RNA, preventing RNA binding proteins from functioning^16^. This steric blocking effect has been leveraged to modulate splicing by blocking the spliceosome, and ASO technology has shown promise in treating patients using ASO drugs such as nusinersen^17,18^ and eteplirsen^19^ to modulate the splicing of target genes. The popularity of ASO technology for treating patients with splicing disorders is exemplified by the successful development of the first patient-customized ASO therapy for Batten’s disease^20^.

Given that many rare disease patients are children, it is crucial to provide accurate diagnoses and develop correct treatments simultaneously. Newborn genome projects such as Genomics England’s generation study^21^ and BabySeq^22^ allow the possibility of developing fast, efficient, and feasible therapeutic approaches tailored to individual mutations. However, it is important to note that not all aberrant splicing in patients can be corrected using ASO technology. The location of the mutation and its proximity to the wild-type splice site play critical roles in the applicability of ASOs^23^. Although patient cell lines are ideal for ASO screening^24^, this option is not feasible for all patients. Although genome editing technologies such as CRISPR-Cas9 exist^25^ to create model systems for ASO screening, they are primarily used for detecting indel mutations rather than substitution mutations^26^, which are prevalent as splicing-disrupting mutations^11^. Therefore, minigenes have been employed as an alternative functional in vitro splicing assay for ASO experiments^27,28^. The current ASO strategy for splicing modulation involves screening multiple ASOs to identify the most potent ASO, a process that can be time-consuming and laborious^29,30^.

We specifically selected five distinct types of aberrant splicing-causing mutations amenable to splicing-modifying ASOs without interfering with wild-type splice sites and designed tailored ASOs to correct aberrant splicing caused by these different mutation types.

## Materials and methods

### Minigene preparation

The pTB vector, spanning 6,003 base pairs (Supplementary data 1), was used as the plasmid for cloning the desired sequences into the NdeI restriction site, which is positioned between the two flanking exons (Supplementary Fig. 3). The detailed sequences of the cloned fragments are provided in Supplementary data 2. The cloning procedures were performed by a professional cloning service provider (GenScript, USA). Site-directed mutagenesis was carried out using PfuUltra II Fusion HotStart DNA Polymerase (Cat No. 600670, Agilent, USA). Plasmid transformation was performed using NEB 5-alpha Competent *E. coli* (high efficiency) cells (NEB, USA), followed by miniprep using the Monarch Plasmid Miniprep Kit (NEB, USA). The plasmid sequencing was conducted by the Sanger Sequencing service company (Source Biosciences, UK).

### Minigene transfection and ASO treatment

The HEK293 cell line is an immortalized human embryonic kidney cell line and an ideal mammalian cell line because of its robustness, rapid growth rate, and ease of maintenance. The cells were used for both minigene transfection and ASO treatment. Approximately 50,000 cells were seeded in individual wells of 24-well plates, each containing 500 µL of Dulbecco’s Modified Eagle’s Medium-high glucose (DMEM) (Sigma‒Aldrich, USA) supplemented with 10% fetal bovine serum (FBS) (Sigma‒Aldrich, USA) and 5% penicillin/streptomycin antibiotic. The seeding was performed one day before the intended transfection. Transfection of cells involved the introduction of 50 ng of wild-type or mutant plasmids per well into DMEM supplemented with 10% FBS and 5% penicillin/streptomycin antibiotic with the aid of Fugene6 transfection reagent (Promega, USA). For minigene analysis, the cells were allowed to incubate for 48 h.

In cases requiring a combination of minigene transfection and ASO treatment, the cells were transfected with plasmids (50 ng per well) using Fugene6 transfection reagent. This step lasted for five hours at 37 °C. Following transfection, the media containing the transfection plasmids was carefully aspirated from each of the wells, followed by a single wash with PBS. Subsequently, 500 µL of Opti-MEM media (Thermo Fisher Scientific, USA) devoid of antibiotics was added to the cells.

The morpholino ASOs were purchased from Gene Tools (Gene Tools, USA), while the 2′-MOE-PS ASOs were purchased from IDT (Integrated DNA Technologies, USA). Standard morpholino, Vivo-morpholino, and 2′-MOE-PS were initially obtained in lyophilized form and subsequently reconstituted using non-DEPC-treated nuclease-free water (AM9330, Thermo Fisher Scientific, USA). Different concentrations of standard morpholino, Vivo-morpholino, and 2′-MOE-PS were prepared in serum-free Opti-MEM media. Standard morpholino solutions were combined with Endo Porter reagent (Gene Tools, USA), facilitating the import of morpholino into the cells. Vivo-morpholino was directly added to the wells without any transfection reagent. Different concentrations of 2′-MOE-PS ASOs were mixed with Lipofectamine RNAiMAX Transfection Reagent (Thermo Fisher Scientific, USA) before being introduced into the cells. All experiments were carried out in triplicate. ASO-treated cells were subsequently incubated at 37 °C before being harvested following a 48-hour treatment period.

### RNA extraction, RT‒PCR, and gel electrophoresis

RNA was extracted from the treated cells using the Qiagen RNeasy Plus Mini Kit (Qiagen, USA). Reverse transcription reactions were performed using a High-Capacity cDNA Reverse Transcription Kit (Thermo Fisher Scientific, USA). Polymerase chain reactions (PCRs) were performed using GoTaq G2 DNA Polymerase (Promega, USA) with primers pTB-FW (TTGATAACCTGAGTCCCGGC) and pTB-RV (TATTTGGAGGTCAGCACGGT) (Integrated DNA Technologies, USA). Gel electrophoresis was carried out on a 3% agarose gel (Sigma‒Aldrich, USA), which was stained with Nancy-520 DNA staining dye (Sigma‒Aldrich, USA) and analyzed using the 50-bp HyperLadder (Meridian Bioscience, USA). The gel documentation process was accomplished utilizing an iBright Imaging System (Thermo Fisher Scientific, USA). Gel band analysis was conducted using iBright analysis software (Thermo Fisher Scientific, USA). Statistical analyses and graphical representations were generated using GraphPad Prism 10.0.2 (Supplementary data 3).

## Results

### Minigenes for aberrant splicing pattern analysis in five different types of mutations

**Fig. 1:**
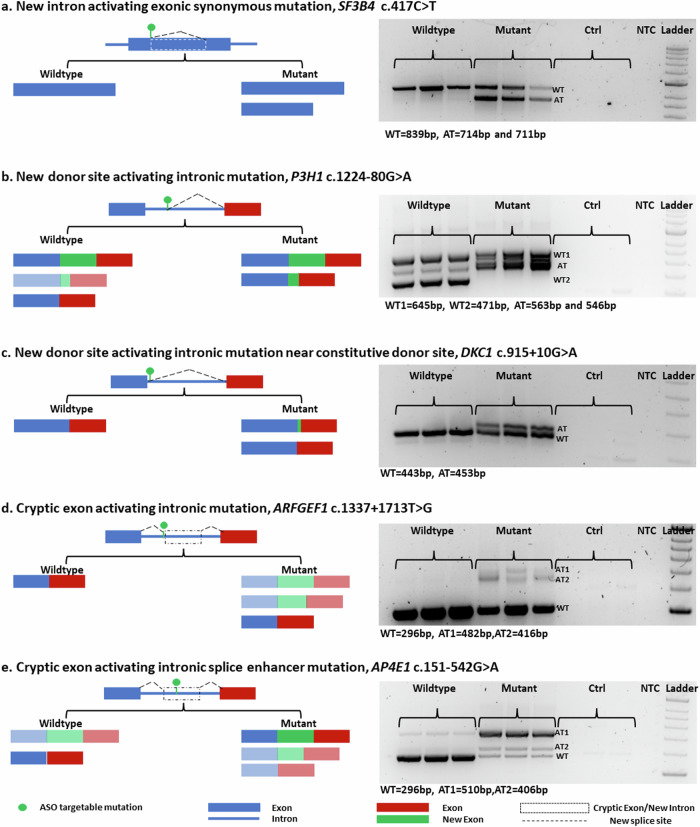
Aberrant splicing patterns in minigenes of five different splicing-disrupting mutation groups in genetic disorders. **a** SF3B4 (c.417C>T): The synonymous exonic mutation of SF3B4c.417C>T introduces a new intron within the exon, resulting in the production of a transcript with partial exon loss in the mutant minigene. **b** The P3H1 minigene produces transcripts with a complete intron retained and spliced in the wild type. However, an intronic P3H1 c.1224-80G>A mutation creates an alternative donor site, leading to a transcript with partial intron retention and loss of the spliced transcript. **c** The intronic DKC1 c.915+10G>A mutation creates an alternative donor site near the wild-type constitutive donor site. In the mutant minigene, both the wild-type and new donor sites are used, resulting in two aberrant transcripts. **d** This intronic mutation, ARFGEF1 c.1337+1713T>G, generates a new acceptor site and activates a new nearby donor site, leading to the generation of cryptic exons in the mutant minigene. **e** The intronic mutation AP4E1 c.151-542G>A functions as an exonic enhancer, leading to the creation of cryptic exons. In the mutant minigene, two cryptic exon transcripts were observed, which were absent in the wild-type minigene.

To investigate the therapeutic potential of ASOs for patients with genetic disorders associated with aberrant splicing, five disease-causing mutations were chosen from our previously published splicing and disease project^11^, each of which represents a different form of clinically encountered aberrant splicing. For every mutation, minigenes and an in vitro splicing test were created and run; RT‒PCR was used to confirm the aberrant splicing pattern (Fig. 1).

#### An exonic *SF3B4* c.417C>T mutation associated with Nager syndrome

This SF3B4 mutation (c.417C>T, NM_005850.5) is located within an exon and creates a new splice donor site and activates a nearby acceptor site within the same exon. This gives rise to a transcript utilizing only part of the exon^11^. The wild-type SF3B4 minigene generates a single transcript that includes the whole exon, and the mutant minigene of SF3B4 generates a transcript with the complete exon and an aberrant transcript with a shortened exon (Fig. 1a). The aberrant splicing pattern produced by the minigene matches that found in the patient’s blood RNA^11^.

#### An intronic *P3H1* c.1224-80G>A mutation associated with osteogenesis imperfecta

The mutation is in an intronic region of *P3H1* (c.1224-80G>A, NM_022356.4), acts as a new donor site^11^, and then is spliced with the nearby acceptor site, which is 80 bp away. Our RT‒PCR analysis of the *P3H1* minigene revealed three transcripts in the wild-type minigene: one band corresponding to the whole intron retained, a faint band with a partial intron retained, and a transcript without an intron (Fig. 1b). On the other hand, the mutant *P3H1* minigene showed a loss of the transcript without introns and increased expression of the transcript with partial retained introns, while the transcript with the whole intron retained remained the same as that of the wild type (Fig. 1b). However, the transcripts of both the entire intron retained and the partial intron retained use alternative acceptor sites; therefore, two transcripts are present in both types. The aberrant splicing pattern produced by the minigene matches that found in the patient’s blood RNA^11^.

#### A proximal splice site mutation in *DKC1*, c.915+10G>A, is associated with dyskeratosis congenita

The mutation generates a new donor site 10 bp away from the wild-type donor site^11^. Our minigene analysis revealed the sole use of the wild-type donor site in the wild-type minigene, with a single transcript, while the mutant minigene showed the use of the wild-type and an aberrant transcript, which uses the new donor site, resulting in a wild-type and an aberrant transcript (Fig. 1c). The aberrant splicing pattern produced by the minigene matches that found in the patient’s blood RNA^11^.

#### An intronic new acceptor mutation of *ARFGEF1*, c.1337+1713T>G, is associated with developmental delay

A mutation in the intron of *ARFGEF1* (c.1337+1713T>G, NM_006421.5) acts as a new acceptor site and activates a new nearby donor site, resulting in cryptic exon generation in patient blood analysis (Supplementary Fig. 1a). Our minigene results revealed cryptic exon activation in the mutant minigene (Fig. 1d). Interestingly, the *ARFGEF1* minigene produces two cryptic exons that use two alternative donor sites. The larger cryptic exon of the aberrant transcript (AT1) is only present in the patient blood analysis, while the minigene produces cryptic exons with both the larger aberrant transcript (AT1) and the smaller aberrant transcript (AT2).

#### An intronic mutation of *AP4E1*, c.151-542G>A, is associated with spastic paraplegia type 51

Unlike the previous intronic mutation, this mutation activates two alternatively spliced cryptic exons. The minigene results revealed activation of two cryptic exons (Fig. 1e), which is consistent with the aberrant splicing pattern found in the patient’s blood. (Supplementary Fig. 1b).

We found that the aberrant splicing minigene patterns matched those in the patient’s blood RNA in four out of five minigenes. A major caveat in minigene construction is the limited size of the gene sequence that can be cloned and inserted into the plasmid. Therefore, it is important to match the aberrant splicing pattern of minigenes to that found in patient samples before proceeding further with ASO design.

### Antisense oligonucleotide treatment to correct aberrant splicing

To correct the aberrant splicing caused by the five different types of individual mutations, we designed tailored ASOs depending on the mutation location and surrounding sequences. We used two main types of ASOs, both of which have been approved for patient treatment: the phosphorodiamidate morpholino oligomer (PMO) and 2′-O-methoxyethyl phosphorothioate (2′-MOE-PS). We also used Vivo-morpholino, which is composed of an octa-guanidylol dendrimer. These results allowed the synthesis of tailored ASOs with three different chemical designs for each mutant and the comparison of the aberrant splicing correction efficacy of standard morpholino, Vivo-morpholino, and 2′-MOE-PS.

### Tailored ASO (YSC-001) correction of aberrant splicing of *SF3B4* c.417C>T mutation associated with Nager syndrome

**Fig. 2:**
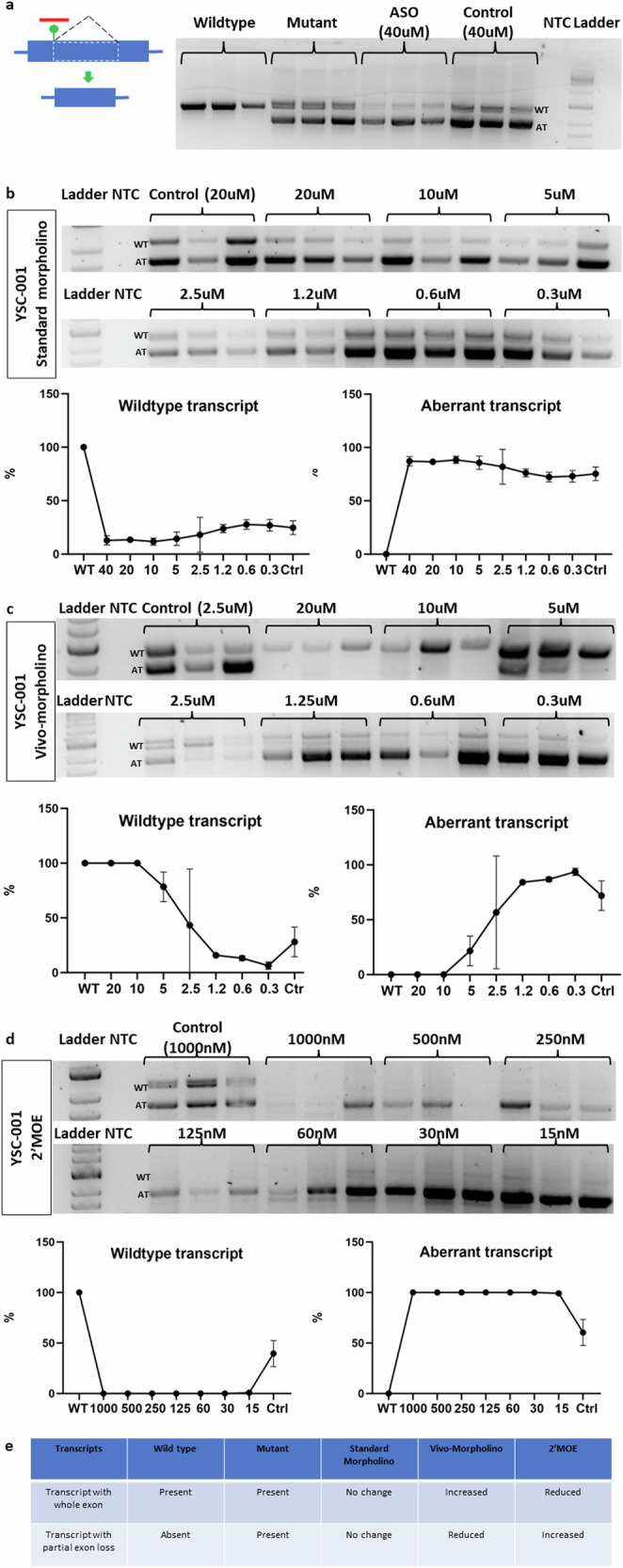
New exonic donor site-activating SF3B4 mutation targeted by YSC-001 via three different chemistries. **a** YSC-001 is specifically designed to target the new donor site, preventing the activation of the new intron. The gel image shows the wild-type and aberrant transcripts in the wild-type minigene, mutant minigene, mutant minigene following standard morpholino treatment (40 µM), and mutant minigene following control morpholino treatment (40 µM). **b** The gel image shows the effects of standard morpholino at different concentrations on the wild-type and aberrant transcripts of the mutant minigene. The graph compares the wild-type and aberrant transcript expression levels of the mutant minigene following ASO treatment to those of the wild-type minigene. **c** The gel image shows the effects of different concentrations of Vivo-morpholino on the wild-type and aberrant transcripts of the mutant minigene. The graph compares the wild-type and aberrant transcript expression levels of the mutant minigene following ASO treatment to those of the wild-type minigene. **d** The gel image shows the effects of 2′MOE at different concentrations on the wild-type and aberrant transcripts of the mutant minigene. The graph compares the wild-type and aberrant transcript expression levels of the mutant minigene following ASO treatment to those of the wild-type minigene. **e** The summary table presents the outcomes of three different types of ASOs with varying chemistries.

An exonic synonymous mutation within *SF3B4* triggers the activation of a new donor site, resulting in the formation of a new intron within the exon. It uses new acceptor sites, resulting in two transcripts in each form. We developed a 25-mer ASO named YSC-001, which is specifically designed to target this mutation (Supplementary Fig. 4a). YSC-001 was engineered to bind to sequences overlapping regions both upstream and downstream of the mutation, effectively obstructing the binding of U1snRNA and spliceosome-associated factors to the new donor site (Fig. 2a). Treatment with standard morpholino, starting at 40 µM and at varying concentrations, failed to correct aberrant splicing (Fig. 2b). However, following Vivo-morpholino treatment starting at a concentration of 20 µM resulted in the complete elimination of aberrant transcripts, with a significant increase in the levels of normal transcripts. Notably, a concentration as low as 5 µM still manifested significant correction, with notable restoration of normal transcripts (Fig. 2c). On the other hand, the 2′-MOE-PS version of YSC-001 had no effect on blocking aberrant splicing at various concentrations (Fig. 2d). Our preliminary experiments on effective concentrations indicate that morpholino effects are observable only at the micromolar (µM) level, whereas 2′-MOE effects can be detected at the nanomolar (nM) level. Interestingly, treatment with YSC-001 2′-MOE blocked the expression of normal transcripts while leaving the aberrant transcript levels unchanged (Fig. 2d). In conclusion, our findings indicate that only the Vivo-morpholino version of YSC-001 effectively blocked the aberrant transcription and facilitated the restoration of the wild-type transcript in the *SF3B4* c.417C>T mutant (Fig. 2e).

### Tailored ASO (YSC-002) correction of aberrant splicing of the *P3H1* c.1224-80G>A mutation associated with osteogenesis imperfecta type VIII

**Fig. 3:**
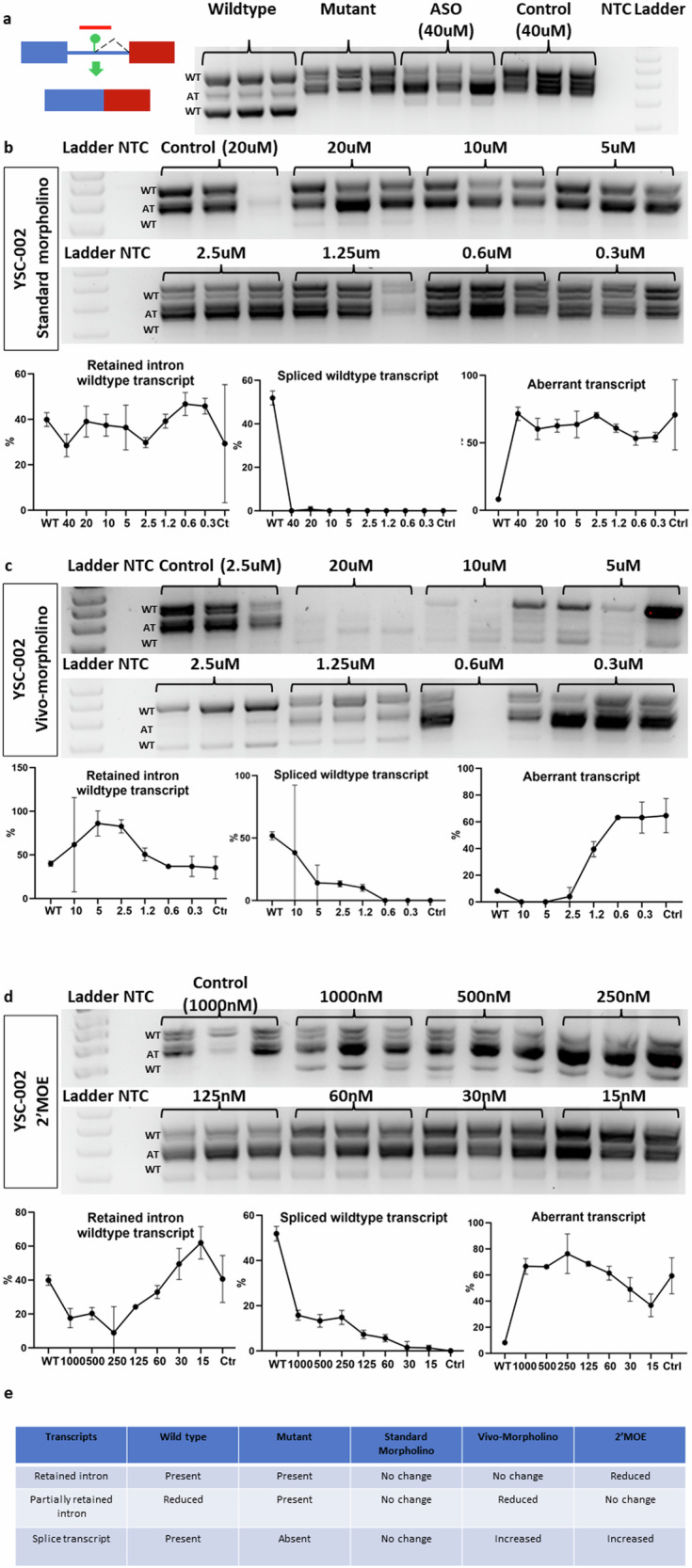
New intronic donor site-activating P3H1 mutation targeted by YSC-002 via three different chemistries. **a** YSC-002 is designed to bind to a new donor site-activating mutant. The gel image shows the wild-type and aberrant transcripts in the wild-type minigene, mutant minigene, mutant minigene following standard morpholino treatment (40 µM), and mutant minigene following control morpholino treatment (40 µM). **b** The gel image shows the effects of standard morpholino at different concentrations on the wild-type and aberrant transcripts of the mutant minigene. The graph compares the wild-type and aberrant transcript expression levels of the mutant minigene following ASO treatment to those of the wild-type minigene. **c** The gel image shows the effects of different concentrations of Vivo-morpholino on the wild-type and aberrant transcripts of the mutant minigene. The graph compares the wild-type and aberrant transcript expression levels of the mutant minigene following ASO treatment to those of the wild-type minigene. **d** The gel image shows the effects of 2′MOE at different concentrations on the wild-type and aberrant transcripts of the mutant minigene. The graph compares the wild-type and aberrant transcript expression levels of the mutant minigene following ASO treatment to those of the wild-type minigene. **e** The summary table presents the outcomes of three different types of ASOs with varying chemistries.

We developed a 25-mer ASO, YSC-002, for a *P3H1* intronic mutation. This ASO was designed to bind both sequences immediately upstream and downstream of the mutation (Supplementary Fig. 4b). At an initial concentration of 40 µM, the ASO treatment did not cause a significant return to the wildtype transcript (Fig. 3a) and became undetectable at 20 µM. Standard morpholino treatment failed to inhibit the formation of the partially retained intron transcript (Fig. 3b). With the Vivo-morpholino version of YSC-002, we observed high cellular toxicity at 20 µM and 10 µM, resulting in lower RNA yields than at other concentrations. Specifically, the 20 µM treatment led to undetectable minigene RNA in the cells. Both the whole retained intron and the spliced transcript reappeared following 10 µM treatment. These effects remained consistent until 2.5 µM, when the expression patterns of the bands closely resembled those of the wild-type minigene (Fig. 3c). At 1.25 µM, all three transcripts were present, but beyond this concentration, the transcript pattern began to resemble that of the untreated mutant minigene. In contrast, the 2′-MOE version of YSC-002 exhibited an increased level of spliced transcripts starting at 1000 nM and maintained detectability across treatments with various concentrations (Fig. 3d). 2′-MOE had no effect on the partially retained intron transcript. Instead, it resulted in a reduced expression level of the whole intron-retaining transcript. In conclusion, our findings suggest that the Vivo-morpholino version of YSC-002 is the most effective ASO (Fig. 3e). Both transcripts with alternative splice sites were treated as a single group for measurement.

### Tailored ASO (YSC-003) correction of aberrant splicing of the *DKC1* c.915+10G>A mutation associated with dyskeratosis congenita

**Fig. 4:**
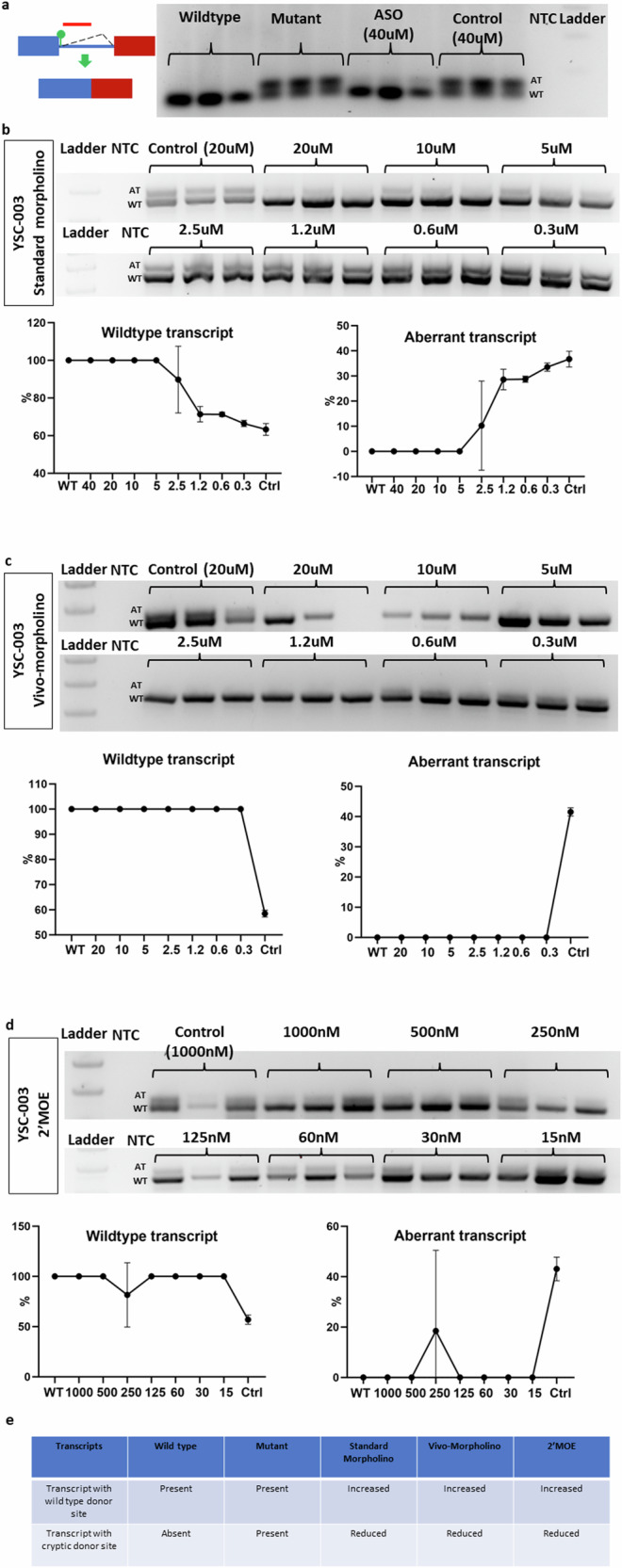
New intronic donor site-activating DKC1 mutation targeted by YSC-003 via three different chemistries. **a** YSC-003 was designed to target a new donor site-activating mutant. The gel image shows the wild-type and aberrant transcripts in the wild-type minigene, mutant minigene, mutant minigene following standard morpholino treatment (40 µM), and mutant minigene following control morpholino treatment (40 µM). **b** The gel image shows the effects of standard morpholino at different concentrations on the wild-type and aberrant transcripts of the mutant minigene. The graph compares the wild-type and aberrant transcript expression levels of the mutant minigene following ASO treatment to those of the wild-type minigene. **c** The gel image shows the effects of different concentrations of Vivo-morpholino on the wild-type and aberrant transcripts of the mutant minigene. The graph compares the wild-type and aberrant transcript expression levels of the mutant minigene following ASO treatment to those of the wild-type minigene. **d** The gel image shows the effects of 2′MOE at different concentrations on the wild-type and aberrant transcripts of the mutant minigene. The graph compares the wild-type and aberrant transcript expression levels of the mutant minigene following ASO treatment to those of the wild-type minigene. **e** The summary table compiles the outcomes of the three different types of chemistries.

We designed YSC-003 differently from previous ASOs (Fig. 4a). In the case of YSC-003, our strategy was to exclusively target the downstream region of the *DKC1* mutation while leaving the upstream region untouched to ensure that the wild-type donor site remained unaffected (Supplementary Fig. 4c). Additionally, we increased the sequence length to 28-mer to enhance the binding affinity. Treatment of the mutant minigene with the standard morpholino version of YSC-003 at 40 µM successfully blocked the aberrant transcript (Fig. 4a). Further experimentation with different concentrations of standard morpholino revealed concentration-dependent blockage of the aberrant transcript, which persisted until reaching 5 µM (Fig. 4b). Similarly, both the Vivo-morpholino and 2′-MOE versions of YSC-003, across various concentration treatments, displayed a variable reduction in aberrant transcripts (Fig. 4c, d). In summary, our findings indicate that all three versions of YSC-003 effectively block the aberrant transcription associated with *DKC1* (Fig. 4e). At a concentration of 20 μM, Vivo-morpholino was toxic, leading to reduced RNA yield.

### Tailored ASO (YSC-004) correction of aberrant splicing of the *ARFGEF1* c.1337+1713T>G mutation associated with developmental delay

**Fig. 5:**
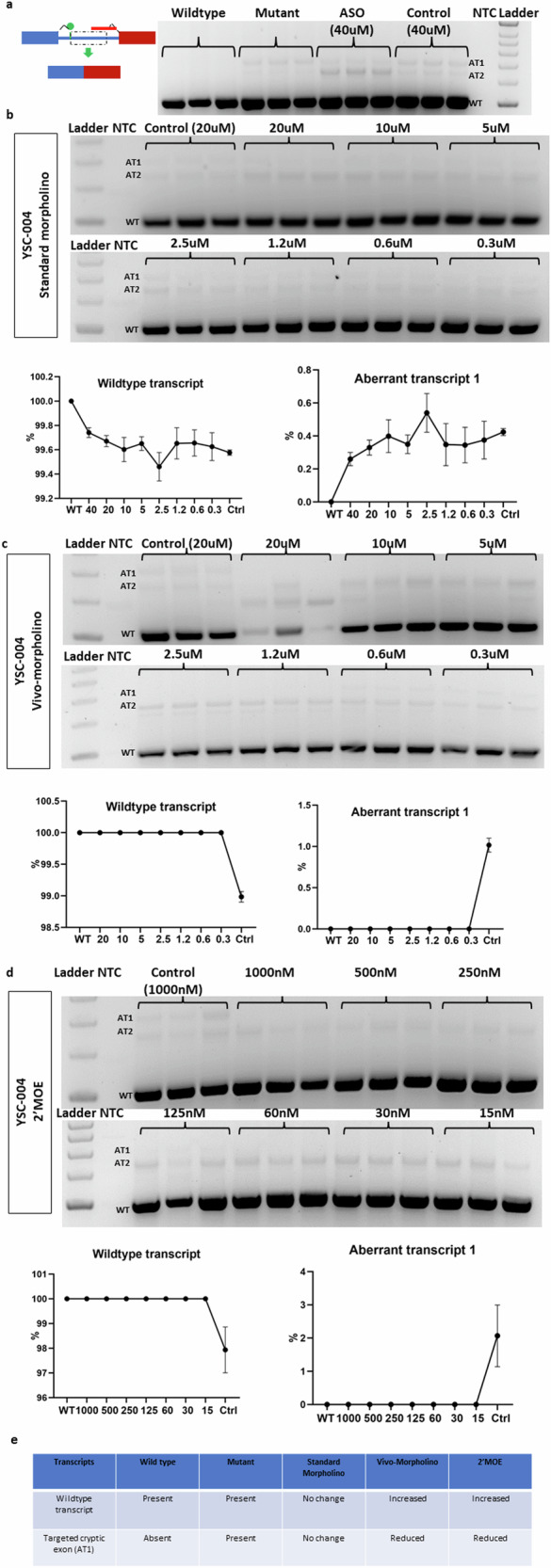
Targeting new intronic donor site-activating ARFGEF1 mutants with YSC-004 using three different chemistries. **a** YSC-004 was designed to target the new donor site of AT1, a cryptic exon-activating mutant. The gel image shows the wild-type and aberrant transcripts (AT1) in the wild-type minigene, mutant minigene, mutant minigene following standard morpholino treatment (40 µM), and mutant minigene following control morpholino treatment (40 µM). **b** The gel image shows the effects of standard morpholino at different concentrations on the wild-type and aberrant transcripts (AT1) of the mutant minigene. The graph compares the wild-type and aberrant transcript expression levels (AT1) of the mutant minigene following ASO treatment to those of the wild-type minigene. **c** The gel image shows the effects of different concentrations of Vivo-morpholino on the wild-type and aberrant (AT1) transcript levels of the mutant minigene. The graph compares the wild-type and aberrant transcript (AT1) expression levels of the mutant minigene following ASO treatment to those of the wild-type minigene. **d** The gel image shows the effects of 2′MOE at different concentrations on the wild-type and aberrant transcripts (AT1) of the mutant minigene. The graph compares the wild-type and aberrant transcript (AT1) expression levels of the mutant minigene following ASO treatment to those of the wild-type minigene. **e** The summary table summarizes the outcomes of three different types of ASOs.

We used a 25-mer ASO, YSC-004, to target the donor site of one cryptic exon transcript (AT1) (Supplementary Fig. 4d) instead of the acceptor site due to the GC-rich nature of the sequence in this area. Treatment of the mutant minigene with the standard morpholino version of YSC-004 at 40 µM reduced the expression of the targeted aberrant transcript (AT1) (Fig. 5a). The band in the wild-type minigene is the flanking exon of the minigene vector. However, the efficiency of cryptic exon blockage diminished as the concentration decreased, becoming evident at 2 µM (Fig. 5b). In contrast, different concentrations of the Vivo-morpholino version of YSC-004 effectively inhibited the targeted transcript (AT1), ranging from 20 µM to 1.2 µM (Fig. 5c). Similarly, treatment with different concentrations of 2′-MOE YSC-004 completely inhibited targeted transcript (AT1) expression (Fig. 5d). In summary, our findings suggest that both Vivo-morpholino and 2′-MOE of YSC-004 ASOs can successfully inhibit the expression of the targeted aberrant transcript (AT1), with 2′-MOE exhibiting the highest efficiency (Fig. 5e).

### Tailored ASO (YSC-005) correction of aberrant splicing in the *AP4E1* c.151-542G>A mutation associated with spastic paraplegia 51 (SPG51)

**Fig. 6:**
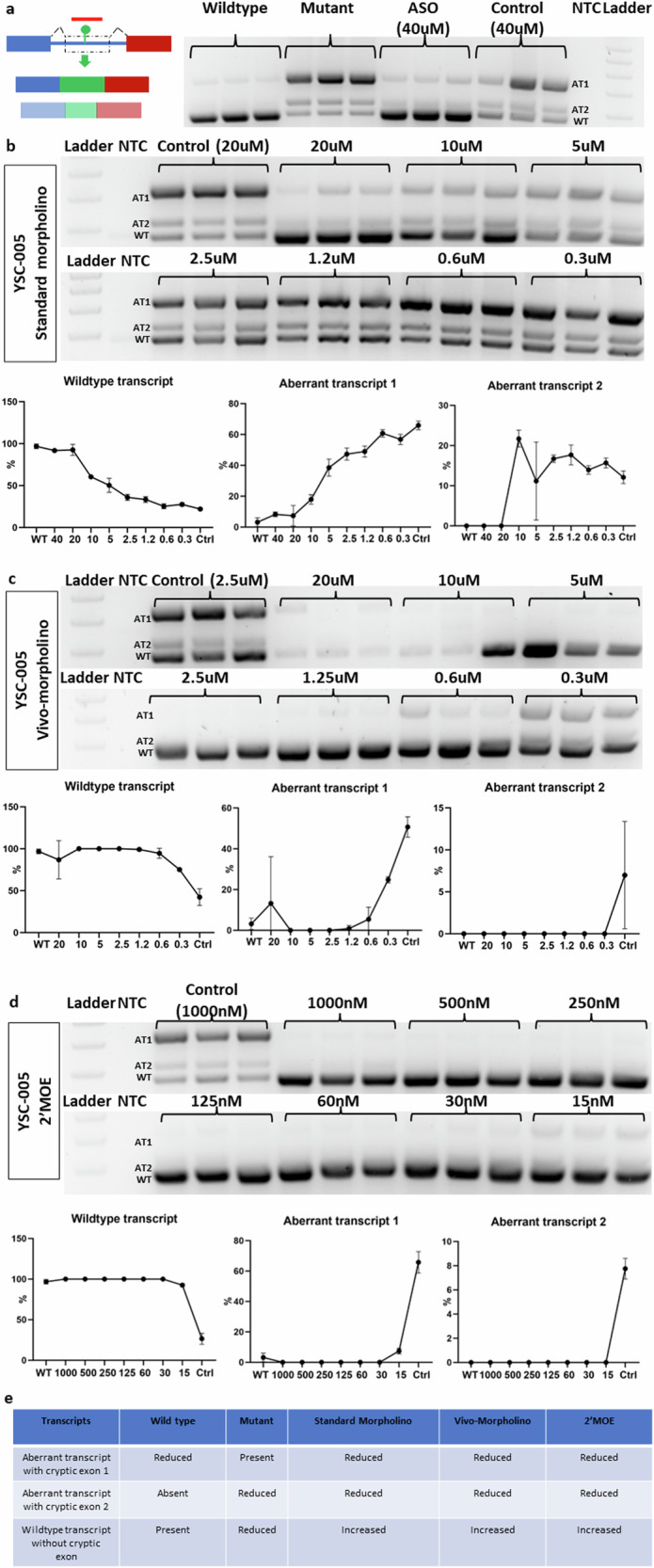
Three different chemistries targeting the intronic cryptic exon that activates the AP4E1 mutation in YSC-005. **a** YSC-005 was designed to target an exonic enhancer mutant. The gel image shows the wild-type and aberrant transcripts in the wild-type minigene, mutant minigene, mutant minigene following standard morpholino treatment (40 µM), and mutant minigene following control morpholino treatment (40 µM). **b** The gel image shows the effects of standard morpholino at different concentrations on the wild-type and aberrant transcripts of the mutant minigene. The graph compares the wild-type and aberrant transcript expression levels of the mutant minigene following ASO treatment to those of the wild-type minigene. **c** The gel image shows the effects of different concentrations of Vivo-morpholino on the wild-type and aberrant transcripts of the mutant minigene. The graph compares the wild-type and aberrant transcript expression levels of the mutant minigene following ASO treatment to those of the wild-type minigene. **d** The gel image shows the effects of 2′MOE at different concentrations on the wild-type and aberrant transcripts of the mutant minigene. The graph compares the wild-type and aberrant transcript expression levels of the mutant minigene following ASO treatment to those of the wild-type minigene. **e** Summary table of the effectiveness of three different types of ASOs. WT = Wild-type transcript without cryptic exons. AT1 and AT2 = Aberrant transcripts with cryptic exons.

We used a 25-mer ASO, YSC-005, to target the enhancer region, encompassing both the upstream and downstream regions flanking the mutation (Supplementary Fig. 4e). The YSC-005 ASO was designed to avoid interference with the donor and acceptor sites of the cryptic exons. Treatment with the standard morpholino version of YSC-005 at a concentration of 40 µM reduced the expression levels of both transcripts containing cryptic exons, accompanied by an increase in the expression of the wild-type transcript devoid of the cryptic exons (Fig. 6a). This effect was consistently observed after treatment with different concentrations of standard morpholino, with cryptic exon inhibition observed until a concentration of 10 µM was reached (Fig. 6b). Similarly, the Vivo-morpholino treatment of YSC-005 ASO inhibited the expression of cryptic exons and restored normal exon expression in a concentration-dependent manner (Fig. 6c). Treatment with various concentrations of the 2′-MOE YSC-005 variant resulted in complete inhibition of both cryptic exons, with efficacy observed at concentrations ranging from 1000 nM to 60 nM (Fig. 6d). In conclusion, all versions of YSC-005 exhibited the ability to restore normal splicing by effectively inhibiting cryptic exons, with 2′-MOE displaying the highest efficiency (Fig. 6e).

These results from five different mutation examples demonstrate the proof of concept for antisense oligonucleotide treatment of actionable splicing-disrupting mutations.

## Discussion

Splicing relies on sequence recognition by the spliceosome and exonic splicing enhancers (ESSs), and the steric blocking effect of ASOs can interfere with this process by directly targeting splicing recognition sequences or exonic splicing enhancers (ESSs). As mentioned, not all mutations causing aberrant splicing are amenable to ASO-based intervention. Mutations that cannot be targeted by ASOs for splicing modification are those that alter the spliceosome recognition motif and disrupt the regular splice sites. These mutations are typically found within physiological splice donors, splice acceptors, branch points, and polypyrimidine track motifs (Supplementary Fig. 2a). Conversely, mutations located away from these splice site regions can be effectively targeted by splice switching ASOs unless they are nonsynonymous exonic mutations. The challenge with nonsynonymous exonic mutations is that they persist in the spliced transcript and may lead to toxic protein production or trigger nonsense-mediated decay.

New splice site-activating synonymous mutations are potential ASO targets, provided they are not situated within or near splice donor or splice acceptor sites (Supplementary Fig. 2b). These mutations can activate new donor or acceptor sites or induce the formation of new introns, such as in the case of the SF3B4 c.417C.T mutation, depending on the nature of the aberrant splicing. Similarly, intronic mutations that activate new splice sites and are distant from wild-type donor, acceptor, branch point, or polypyrimidine track motifs can also be effectively targeted by ASOs. This category encompasses new acceptor or donor site-activating mutations, resulting in retained intron transcripts such as that observed with DKC1 c.915+10G>A (Supplementary Fig. 2c), as well as those activating new donor/acceptor sites, including cryptic exons (Supplementary Fig. 2d), such as that observed with P3H1 c.1224-80G>A, ARFGEF1 c.1337+1713T>G, and AP4E1 c.151-542G>A. Additionally, mutations functioning as exonic enhancers can activate cryptic exons and are suitable targets for ASOs (Supplementary Fig. 2e). While mutations near splice site regions pose challenges for ASO targeting, they can still be selectively addressed by meticulously designed ASOs that do not disrupt wild-type splice sites (Supplementary Fig. 2f).

In this study, we tested 5 disease-causing mutations belonging to the classes mentioned in S1 B through F and demonstrated that mutations located away from wild-type splicing regulatory motifs are amenable to correction using splicing-modifying ASOs. Furthermore, we have shown that ASOs can effectively target mutations near the wild-type splice site without causing interference with the adjacent splice site.

Currently, there is no database specifically dedicated to actionable splicing-disrupting mutations. Estimating the prevalence of such mutations in existing databases poses a particular challenge, as databases such as ClinVar often do not categorize alterations in or near splice sites as pathogenic, especially when the mutation does not alter the sequence of the encoded protein^31^. In our previous study, where we analyzed aberrant splicing in patient blood RNA, we found that approximately 27% of all splice site-affecting mutations were alternative donor site splicing mutations, making them potential targets for antisense oligonucleotides^11^.

Our findings also highlight the potential need for multiple ASOs to treat specific patients. For instance, in our analysis of the *ARFGEF1* minigene, which produces two cryptic exons triggered by a single new acceptor site-activating mutation, we encountered a unique challenge. Targeting the new acceptor site was hindered by a cytosine-rich sequence near this site, as oligonucleotides designed to target such regions could form G-quadruplexes, leading to reduced oligonucleotide binding efficiency. Therefore, we opted to target the donor site within the cryptic exon. While this approach effectively blocked one cryptic exon, the presence of the other remained unaffected.

The current estimated timeframe for ASO development before patients begin treatment through the n-Lorem Foundation recruitment process is 12 to 15 months^32^. However, an accelerated timeframe is possible. The process from splice defect identification to ASO functionality validation in Milasen drug development, involving the screening of seven different ASO sequence designs, was achieved in only five months^20^. Our approach involves screening three different chemistries to correct aberrant splicing using a minigene assay in the HEK203 cell line, a process that can be completed in less than two months. However, it is crucial to validate ASO functionality in cell types relevant to disease phenotypes. While the HEK293 cell line is versatile and easy to maintain, many other cell lines pose challenges in maintenance and exhibit slow growth rates. Therefore, selecting appropriate cell lines for testing ASO functionality will be a critical aspect of rapid ASO screening.

Our investigation also revealed the significant influence of chemical design on the efficiency of aberrant splicing rescue. Notably, we demonstrated that aberrant splicing correction can be transcript-specific. In our experiment involving the YSC-002 ASO on the P3H1 minigene, different chemical designs exhibited preferences for specific transcript inhibition. While the majority of our ASOs were designed to target aberrant donor sites, we observed varying efficacies among the different ASO types, suggesting that certain chemical designs possess a propensity for binding to particular sequences. We also noted the remarkable and contrasting effects of 2’-MOE YSC-002 on the P3H1 mutation minigene, where it enhanced splicing in the spliced transcript while inhibiting splicing in the retained intron transcript. This effect may be caused by the ASO remaining bound to the RNA and recruiting intracellular proteins to induce degradation^33^. Thus, our results highlight the critical role of chemistry in ASO development for therapeutic applications.

Our study further revealed cases in which all three ASO classes effectively rescued aberrant splicing, such as in the instances of *DKC1*, *AP4E1*, and *ARFGEF1* mutations. These findings provide valuable flexibility, allowing the choice of the most suitable ASO chemistry depending on the target cell, tissue, or administration route. Notably, PMO class ASOs do not bind to any proteins, resulting in a relatively short plasma half-life^34,35^. In contrast, 2′-MOE ASOs exhibit a longer tissue elimination half-life^36^. The rescue of aberrant splicing by both ASO classes enables the selection of the most desirable chemistry based on the specific requirements of the target system. While both Vivo-morpholino and 2′-MOE demonstrate promising efficacy, it is also crucial to consider ASO toxicity^37,38^ and carry out toxicology studies of specific ASOs on relevant cells and tissues before proceeding to clinical trials.

In conclusion, our results highlight the potential of single, tailored ASO designs to effectively rescue aberrant splicing across various target groups. This finding holds significant promise for the development of rapid, efficient, and scalable preclinical therapeutic strategies, addressing the pressing need for rare disease therapeutics. As newborn screening initiatives such as BabySeq and Genomics England’s newborn genome sequencing have gained traction, our ASO results offer hope to patients and their families, ensuring timely access to the personalized treatment.

## Supplementary information

Supplementary Information

Supplementary data 3
