# Supplementary material for: Tailored antisense oligonucleotides designed to correct aberrant splicing reveal actionable groups of mutations for rare genetic disorders
Source: Exp Mol Med. 2024 Aug 1;56(8):1816–25. doi: 10.1038/s12276-024-01292-1 (PMC11371919; doi:10.1038/s12276-024-01292-1)
Supplement: Supplementary file 1 — Supplementary Information [file 12276_2024_1292_MOESM1_ESM.pdf]

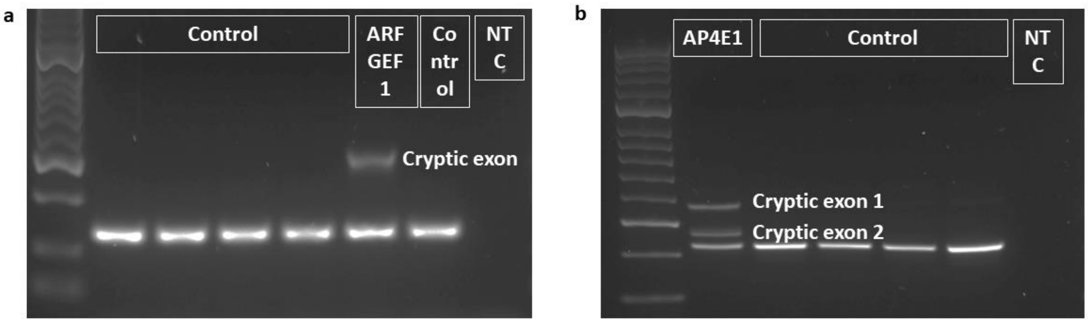

Supplementary figure 1. RT-PCR gel electrophoresis picture of ARFGEF1 c.1337+1713T>G and AP4E1 c.151-542G>A using patient blood samples. (a) ARFGEF1 variant causes the activation of a cryptic exon in the patient blood sample. (b) AP4E1 intronic variant induces two cryptic exons in the patient blood sample.

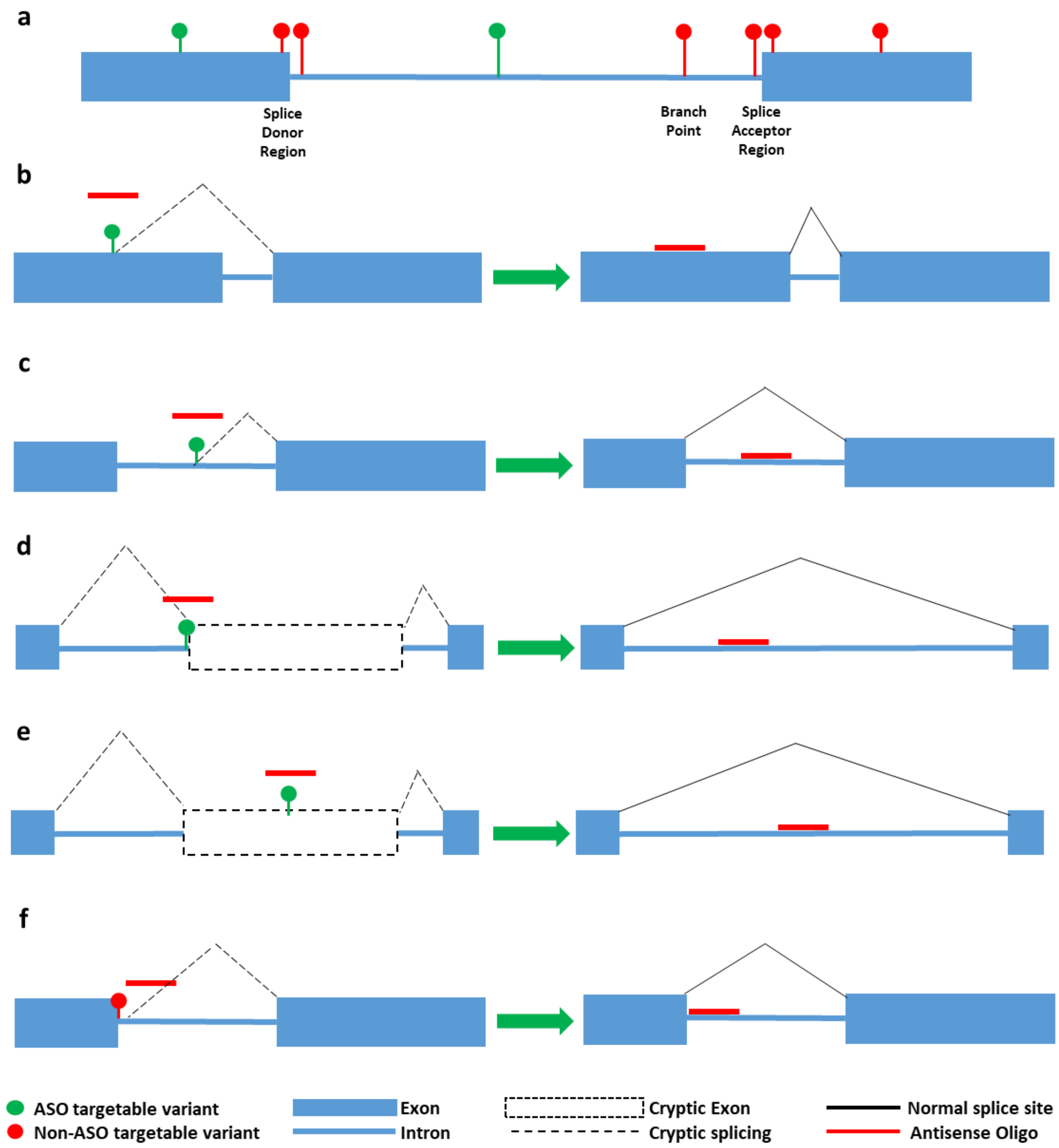

Supplementary figure 2. Determining ASO targetability of aberrant splicing variants. (a) ASO targetable variants are generally located in the region far away from splice donor and acceptor sites. (b) Cryptic splice site activating exonic synonymous variants can be targeted by ASO to retore the normal splicing. (c) Cryptic splice site activating intronic variant can also be targeted by ASO for splicing correction. (d) Variant activating donor site of cryptic exon can also be targeted by ASO or (e) Cryptic exon activating distant variant can be silenced with ASO by directly masking the variant region without interfering the cryptic exon (f) Variant near the splice donor site can be targeted without interfering the constitutive splice donor site.

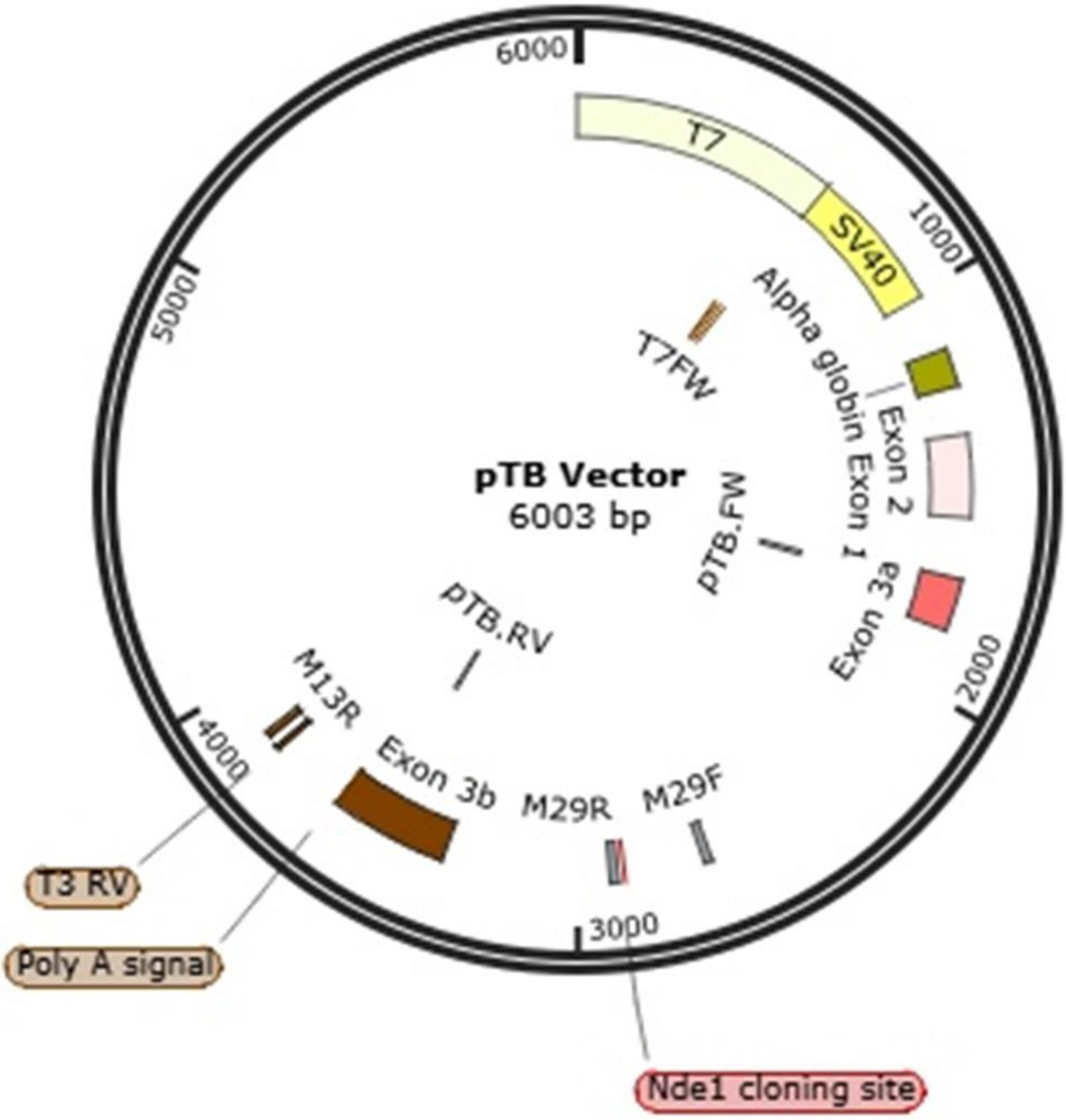

Supplementary figure 3. Map of pTB vector plasmid. The vector is 6003bp long and it has four exons. NdeI cloning site was located between Exon 3a and Exon 3b.

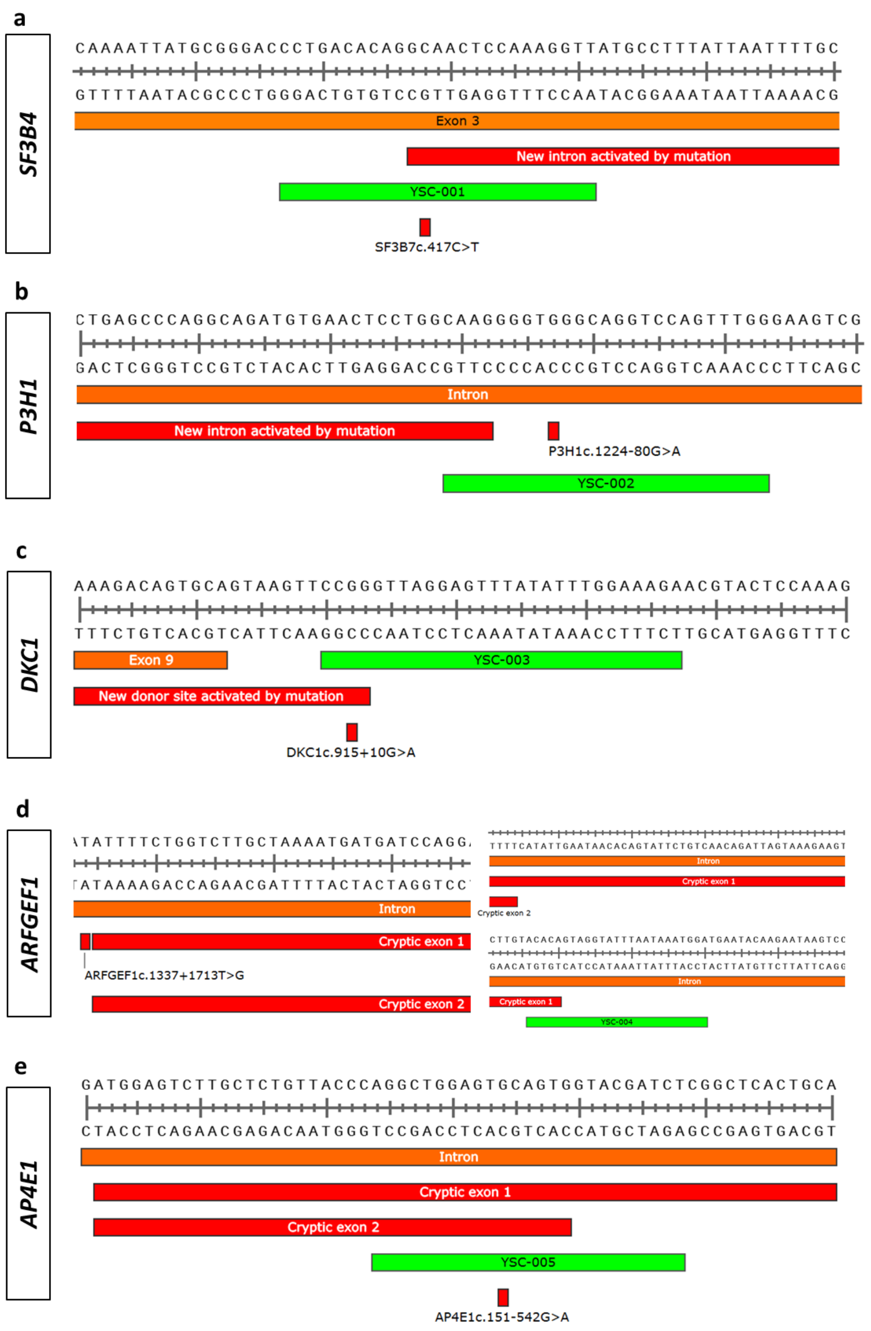

Supplementary figure 4. Genomic sequences with mutations, aberrant splicing and ASO target sequence locations. (a) The synonymous exonic mutation of SF3B4c.417C>T activates a new donor site which was targeted with YSC-001 (AACCTTTGGAGTTACCTGTGTCAGG). (b) The P3H1 c.1224-80G>A mutation creates a new donor site which was targeted with YSC-002 (CCAAACTGGACCTGCCTACCCCTTG). (c) The intronic DKC1 c.915+10 G>A mutation generates a new donor site near the wild-type constitutive donor site and was targeted with YSC-003 (TCTTTCCAAATATAAACTCCTAACCTGG). (d) This intronic mutation ARFGEF1 c.1337+1713T>G activates a new acceptor site and activates two cryptic donor sites, and one donor site was targeted with YSC-004 (TCCATTTATTAAATACCTACTGTGT). (e) The intronic mutation AP4E1 c.151-542G>A functions as an exonic enhancer, leading to the creation of two cryptic exons. The enhancer site was targeted with YSC-005 (AGATCGTACCACTGTACTCCAGCCT).

Supplementary data 1

pTB Vector Sequence

CTAAATTGTAAGCGTTAATATTTTGTTAAAATTCGCGTTAAATTTTTGTTAAATCAGCTCATTTTTTAACC
AATAGGCCGAAATCGGCAAAATCCCTTATAAATCAAAAGAATAGACCGAGATAGGGTTGAGTGTTGTT
CCAGTTTGGAACAAGAGTCCACTATTAAAGAACGTGGACTCCAACGTCAAAGGGCGAAAAACCGTCT
ATCAGGGCGATGGCCCACTACGTGAACCATCACCCTAATCAAGTTTTTTGGGGTCGAGGTGCCGTAAA
GCACTAAATCGGAACCCTAAAGGGAGCCCCCGATTTAGAGCTTGACGGGGAAAGCCGGCGAACGTG
GCGAGAAAGGAAGGGAAGAAAGCGAAAGGAGCGGGCGCTAGGGCGCTGGCAAGTGTAGCGGTCA
CGCTGCGCGTAACCACCACACCCGCCGCGCTTAATGCGCCGCTACAGGGCGCGTCCCATTCGCCATTC
AGGCTGCGCAACTGTTGGGAAGGGCGATCGGTGCGGGCCTCTTCGCTATTACGCCAGCTGGCGAAAG
GGGGATGTGCTGCAAGGCGATTAAGTTGGGTAACGCCAGGGTTTTCCCAGTCACGACGTTGTAAAAC
GACGGCCAGTGAGCGCGCGTAATACGACTCACTATAGGGCGAATTGGAGCTCGGTACCCCTGTGGAAT
GTGTGTCAGTTAGGGTGTGGAAAGTCCCCAGGCTCCCCAGCAGGCAGAAGTATGCAAAGCATGCATC
TCAATTAGTCAGCAACCAGGTGTGGAAAGTCCCCAGGCTCCCCAGCAGGCAGAAGTATGCAAAGCAT
GCATCTCAATTAGTCAGCAACCATAGTCCCGCCCCTAACTCCGCCCATCCCGCCCCTAACTCCGCCCAGT
TCCGCCCATTCTCCGCCCCATGGCTGACTAATTTTTTTTATTTATGCAGAGGCCGAGGCCGCCTCGGCCT
CTGAGCTATTCCAGAAGTAGTGAGGAGGCTTTTTTGGAGGCCTAGGCTTTTGCAAAAAGCTCCAAGC
TCTCGAGGATCCcccgggctccgcgccagccaatgagcgccgcccggccgggcgtgcccccgcgccccaagcataaaccctgg
cgcgctcgcggcccggcactcttctggtccccacagactcagagagaacccaccATGGTGCTGTCTCCTGCCGACAAGAC
CAACGTCAAGGCCGCCTGGGGTAAGGTCGGCGCGCACGCTGGCGAGTATGGTGCGGAGGCCCTGGA
GAGgtgaggctccctcccctgctccgacccgggctcctcgcccgcccggacccacaggccaccctcaaccgtcctggccccggacc
caaaccccacccctcactctgcttctccccgcagGATGTTCCTGTCCTTCCCCACCACCAAGACCTACTTCCCGCAC
TTCGACCTGAGCCACGGCTCTGCCCAGGTTAAGGGCCACGGCAAGAAGGTGGCCGACGCCCTGACC
AACGCCGTGGCGCACGTGGACGACATGCCCAACGCGCTGTCCGCCCTGAGCGACCTGCACGCGCACA
AGCTTCGGGTGGACCCGGTCAACTTCAAGgtgagcggcgggccgggagcgatctgggtcgaggggcgagatggcgcc
ttcctcgcagggcagaggatcacgcgggttgcgggaggtgtagcgcaggcggcggctgcgggcctgggccctcggccccactgacc
ctcttctctgcacagCTCCTAAGCCACTGCCTGCTGGTGACCTGCACTTTTGATAACCTGAGTCCCGGCCTGG
AGTACAATGTCAGTGTTTACACTGTCAAGGATGACAAGGAAAGTGTCCCTATCTCTGATACCATCATCCC
AGgtaatagaataaataagctgctatcctgagagtgacacttccaataagagtggggattagcatcttaatccccagatgcttaagg
gtgtcaactatatttgggatttaattccgatctcccagctgcactttccaaaaccaagaagtcaaagcagcgatttggacaaatgcttgc
tgttaacactgctttactgtctgtgcttcactgggatgctgtgtgttgcagcgagtatgtaatggagtggcagccatggctttaactctgt
attgtctgctcacatggaagtatgactaaaacactgtcacgtgtctgtactcagtactgataggctcaaagtaatatggtaaatgcatc
ccatcagtacatttctgcccgattttacaatccatatcaatttccaacagctgcctataaaatagttttgtccctgtatgtgagcactgaaa
cagcatttggttgacacatctagtttttcatcttgcagtttcaaatccttctttttgaaaattggatttaaaaaaaagaagtaaaagtcac
accttcagggtgttctttcttgtggcttgaaagacaacattgcaaaggcctgtctaaggataggcttgtttgtccattgggttataacata
atgaaagcattggacagatcgtgtcccccttggactcttcagtagaatgcttttactaacgctaattacatgttttgattatgaatgaact
aaaatagtggcaatggccttaaccttaggcctgtctttcctcagcctgaatgtgcttttgaatggcacatttcacaccatacattcataat
gcattagcgttatggccatgatgttgtcatgagttttgtatgggagaaaaaaaatcaatttatcacccatttattatttttaaccttcgttc
atgcaagcttatttctactaaaacagttttggaattattaaaagcattgctgatacttacttcagatattatgtctaggctctaagaatgg
tttcgacatcctaaacagccaccaccgcggtggcggccccatatggtcgagggggggcccgctagatatctcacatgggggaagtt
taggaccctcttgtctttttgtctgtgtgcatgtatttctttgtaaagtactgctatgtttctctttgctgtgtggcaacttaagcctcttcggc
ctgggataaaataatctgcagtggtattaataatgtacataaagtcaacatatttgaaagtagattaaaatcctttttaaatatatcaat
gatggcaaaaaggttaaagggggcctaacagtactgtgtgtagtgttttattttaacagtagtacactataacttaaaatagacttag

attagactgtttgcatgattatgattctgtttcctttatgcatgaaatattgattttacctttccagctacttcgttagctttaattttaaaatta
cattaactgagtcttccttcttgttcgaaaccagCTGTTCCTCCTCCCACTGACCTGCGATTCACCAACATTGGTCCA
GACACCATGCGTGTCACCTGGGCTCCACCCCCATCCATTGATTTAACCAACTTCCTGGTGACCCTGGCC
GCCCACCTCCCCGCCGAGTTCACCCCTGCGGTGCACGCCTCCCTGGACAAGTTCTGCTTCTGTGAGCA
CCGTGCTGACCTCCAAATACCGTTAAGCTGGAGCCTCGGTGGCCATGCTTCTTGCCCCTTGGGCCTCCC
CCCAGCCCCTCCTCCCCTTCCTGCACCCGTACCCCCGTGGTCTTtgaataaagtctgagtgggcggcagcctgtgt
gtgcctgagtttttccctcagcaaacgtgccaggcatgggcgtggacagcagctgggacacacatggctagaaCCTCTCTGCAG
GAATTCGATATCAAGCTTATCGATACCGTCGACCTCGAGGGGGGGCCCGGTACCCAGCTTTTGTTCCCT
TTAGTGAGGGTTAATTGCGCGCTTGGCGTAATCATGGTCATAGCTGTTTCCTGTGTGAAATTGTTATCCG
CTCACAATTCCACACAACATACGAGCCGGGAGCATAAAGTGTAAAGCCTGGGGTGCCTAATGAGTGAG
CTAACTCACATTAATTGCGTTGCGCTCACTGCCCGCTTTCCAGTCGGGAAACCTGTCGTGCCAGCTGCA
TTAATGAATCGGCCAACGCGCGGGGAGAGGCGGTTTGCGTATTGGGCGCTCTTCCGCTTCCTCGCTCA
CTGACTCGCTGCGCTCGGTCGTTCGGCTGCGGCGAGCGGTATCAGCTCACTCAAAGGCGGTAATACG
GTTATCCACAGAATCAGGGGATAACGCAGGAAAGAACATGTGAGCAAAAGGCCAGCAAAAGGCCAG
GAACCGTAAAAAGGCCGCGTTGCTGGCGTTTTTCCATAGGCTCCGCCCCCCTGACGAGCATCACAAAA
ATCGACGCTCAAGTCAGAGGTGGCGAAACCCGACAGGACTATAAAGATACCAGGCGTTTCCCCCTGG
AAGCTCCCTCGTGCGCTCTCCTGTTCCGACCCTGCCGCTTACCGGATACCTGTCCGCCTTTCTCCCTTCG
GGAAGCGTGGCGCTTTCTCATAGCTCACGCTGTAGGTATCTCAGTTCGGTGTAGGTCGTTCGCTCCAA
GCTGGGCTGTGTGCACGAACCCCCCGTTCAGCCCGACCGCTGCGCCTTATCCGGTAACTATCGTCTTGA
GTCCAACCCGGTAAGACACGACTTATCGCCACTGGCAGCAGCCACTGGTAACAGGATTAGCAGAGCG
AGGTATGTAGGCGGTGCTACAGAGTTCTTGAAGTGGTGGCCTAACTACGGCTACACTAGAAGGACAGT
ATTTGGTATCTGCGCTCTGCTGAAGCCAGTTACCTTCGGAAAAAGAGTTGGTAGCTCTTGATCCGGCA
AACAAACCACCGCTGGTAGCGGTGGTTTTTTTGTTTGCAAGCAGCAGATTACGCGCAGAAAAAAAGG
ATCTCAAGAAGATCCTTTGATCTTTTCTACGGGGTCTGACGCTCAGTGGAACGAAAACTCACGTTAAG
GGATTTTGGTCATGAGATTATCAAAAAGGATCTTCACCTAGATCCTTTTAAATTAAAAATGAAGTTTTAA
ATCAATCTAAAGTATATATGAGTAAACTTGGTCTGACAGTTACCAATGCTTAATCAGTGAGGCACCTATCT
CAGCGATCTGTCTATTTCGTTCATCCATAGTTGCCTGACTCCCCGTCGTGTAGATAACTACGATACGGGA
GGGCTTACCATCTGGCCCCAGTGCTGCAATGATACCGCGAGACCCACGCTCACCGGCTCCAGATTTATC
AGCAATAAACCAGCCAGCCGGAAGGGCCGAGCGCAGAAGTGGTCCTGCAACTTTATCCGCCTCCATC
CAGTCTATTAATTGTTGCCGGGAAGCTAGAGTAAGTAGTTCGCCAGTTAATAGTTTGCGCAACGTTGTT
GCCATTGCTACAGGCATCGTGGTGTCACGCTCGTCGTTTGGTATGGCTTCATTCAGCTCCGGTTCCCAA
CGATCAAGGCGAGTTACATGATCCCCCATGTTGTGCAAAAAAGCGGTTAGCTCCTTCGGTCCTCCGATC
GTTGTCAGAAGTAAGTTGGCCGCAGTGTTATCACTCATGGTTATGGCAGCACTGCATAATTCTCTTACTG
TCATGCCATCCGTAAGATGCTTTTCTGTGACTGGTGAGTACTCAACCAAGTCATTCTGAGAATAGTGTAT
GCGGCGACCGAGTTGCTCTTGCCCGGCGTCAATACGGGATAATACCGCGCCACATAGCAGAACTTTAA
AAGTGCTCATCATTGGAAAACGTTCTTCGGGGCGAAAACTCTCAAGGATCTTACCGCTGTTGAGATCC
AGTTCGATGTAACCCACTCGTGCACCCAACTGATCTTCAGCATCTTTTACTTTCACCAGCGTTTCTGGGT
GAGCAAAAACAGGAAGGCAAAATGCCGCAAAAAAGGGAATAAGGGCGACACGGAAATGTTGAATAC
TCATACTCTTCCTTTTTCAATATTATTGAAGCATTTATCAGGGTTATTGTCTCATGAGCGGATACATATTTG
AATGTATTTAGAAAAATAAACAAATAGGGGTTCCGCGCACATTTCCCCGAAAAGTGCCAC

Supplementary data 2

>SF3B4

tctcttcagtttatttctggaaccattcttaagtaaagtgatgttgtttctttcttgttctttgtgattagagggtagatgaattttaccccattt
tcagtctctttacttacgttgttaacctcctcttctccacttttccagGCTATGGCTTTGTGGAATTCTTGAGTGAGGAAGA
TGCTGACTATGCCATTAAGATCATGAACATGATCAAACTCTATGGGAAGCCAATACGGGTGAACAAAGC
ATCAGCTCACAACAAAAACCTGGATGTAGGGGCCAACATTTTCATTGGGAACCTGGACCCTGAGATTG
ATGAGAAGTTGCTTTATGATACTTTCAGCGCCTTTGGGGTCATCTTACAAACCCCCAAAATTATGCGGG
ACCCTGACACAGGCAACTCCAAAGGTTATGCCTTTATTAATTTTGCTTCATTTGATGCTTCGGATGCAGC
AATTGAAGCCATGAATGGGCAGTACCTCTGTAACCGTCCTATCACCGTATCTTATGCCTTCAAGAAGGA
CTCCAAGGGTGAGCGCCATGGCTCAGCAGCCGAACGACTTCTGGCAGCTCAGAACCCGCTCTCCCAG
GCTGATCGCCCTCATCAGCTGTTTGCAGATGCACCTCCTCCACCCTCTGCTCCCAATCCTGTGGTATCAT
CATTGGGGTCTGGGCTTCCTCCACCAGgtaaagcttttggttaaaatatgtttgtcttaaggttgggggagagggtcagga
ggaagaagaaaacaagtctgaaaggggggacaggggacattgagtcagtgagttgatgggaagaggactgaggggtgatggtagt
ggtgaagaagaaagttgttgggtttctttaggagggtgaagttggttgactttctaacattgcttttgatttagagcactggg

>P3H1

ggaagagggtgataagctggattactcctgtgggccttattttatgcattgagacacctggaagattaagtagatcatcttaccttttca
tttcttcctgacctacttagGATTCATGGACTCCAGAAGAAGTGATTCCCAAGAGATTGCAAGAGAAACAGAA
gtgaggaccttgaagaaactgcatggttggatcagtctgatgaagcacttgaggcttcctgagcccaggcagatgtgaactcctggca
aggggtgggcaggtccagtttgggaagtcggggtggagcccagggctggccctggaatgcagtcctcagagcggctgtgctcatag
GTCAGAACGGGAAACAGCCGTACGCATCTCCCAGGAGATTGGGAACCTTATGAAGGAAATCGAGACC
CTTGTGGAAGAGAAGACCAAGGAGTCACTGGATGTGAGCAGACTGACCCGGGAAGgtgagaggacaga
aataggggcatgtctggctgttgtcagggagccaggcaggtcaccccacatttgccag

>DKC1

aagagccaggactgaagaggctgaagacataatgagcttggactagtaaacaagtgacattcagttttaggaagtttggggtctgat
gggctgagatacaaataaactgaattattttcatacatggctgcttttcagGACCACATGGTGACAATGCATGATGTGCTT
GATGCTCAGTGGCTGTATGATAACCACAAGGATGAGAGTTACCTGCGGCGAGTTGTTTACCCTTTGGA
AAAGCTGTTGACATCTCATAAACGGCTGGTTATGAAAGACAGTGCAgtaagttccgggttaggagtttatatttgg
aaagaacgtactccaaagatgaggcttgctctttttattgttttccttgggttttattttattttttcaagtccaaaccaagtatattaattta
aagtaaaagtttaatggtaggcatacttaaaaataaggcacattaggccaggcacagtggctgatgcctataatcctagcactttggc
aggctgag

>ARFGEF1

attgctgaagggcattttttgccatgctttaaaaatgttattgaccattaaagcctcagatgctttttttccaaatcaaatgtaaaaattgtga
tttgaaagaatgctagaaacagaaaagaagaaattctttaaaatgctgagaatgaaaaggacacaaaggaaaataatatgaatttt
gaggagtatgttcatttattcctgaaaaaaagagtgatctggttttgaaaagatgctatctttaatgttacaaaggattttatgaggaat
gatgtaaattaacaagccttatacttagtgggtttaaaccaaatattaggaaattttgtttgaagaccttcttgacttgactgtcagggtc
attaaacaatggaattgaatactgaatacacccttagaagctattattttccaaaagaatatgtagttttctgttttaatgattttttttttt
tttgccaaaagtcaagctttcgtagttacatcattttctttttttgtctttgcttccccttatattttctggtcttgctaaaatgatgatccagg
aaatgttcttgaagcctgaatcataatttgaaactaccccatgaaaaactgcagacctgaaatttccagcctcttttgcctatcaaaagt
ataacttattgtgtcataagacagttgtctaatcatttcttcattcattattgccttgtacacagtaggtatttaataaatggatgaatacaa
gaataagtccattaattttttttagcatgtacagtgtacaagatattttgttaaatgttgggaatacaaatataaaacatacttagaacaa

acactcattgttatagaatggtaagctgcaggatttaattacaataggaattgagcataataggtgataaatgtgagctagaatagga
agtagtgtgtaagccttggaagactagaacaggaggggaggtggtgggttaatttgtagttccctgtagctatttgaagtcttagttctt
gctgtctctacttgatttatctaagcatttttttggtaattatctatttgctgcatatattcttagattcaaaggaagactcagcacaatggc
catcttagaaacatgactgtgtaataatgagccataatattattggctccagttttatttcagagaaagtagtattttttgtgatacccatt
tacgtgaaattaacaattccttattagatttacgtagcttcttgaattacttgacactta

>AP4E1

gataggcatccacgtgtatcagtacgtcactagtggcttttgaatagccatctgatgaacatgtaattcagtgtgtaatttaaaggtagc
atgatattagccaaaaaaaaaatggggccaatttttaagtgcttagggagataacatacaatgcagacagtaggagtgaccacattg
gtatgtgtctgagataggaagttatggaaaggatttggggaatgaggaaaagacagggaagaagaatgtagtttaagggaaaaag
acctatttctgtttctgctttcttaccctgtttcttaattccatgaagacttttacttctaaggggaagatgtgagatgagactcttttcttgg
gccaatacatggagaatttctactctctctccacctttaatttttttttttttttttttagatggagtcttgctctgttacccaggctggagt
gcagtggtacgatctcggctcactgcaacattcacttcccgggttcaagcgattctcctgcccctgcctcagcctctcgagtatctggga
ttacaggacccccagctaatgtttgtatttttagtagggacggggttttgccatgttggccaggctggtctcaaactgctgacctcaagt
gattcactcgccttggcctcccaaagtgctgggattacaggcatgagctgttgtgcccggcttccacctttagttttaaaaaaaaacattt
tctgctattctaccattaaggggaagtaactgagtaaagaaaagaggaaggatgggttagcctggaaatatttattctctccagggat
ggttctgaggtttggagaaatgcctttagatagatcgtaagcagatagtatttgtaactctcctttaaaataatgtttcttttaaattaaaa
gtattgtaaattatatttttccgtttagataaatgctgttacagttttaaaagacagttaatcaagcatttaaatattttcactttctcagga
agaagaaaaattaatccagcaggaactgagtagtctgaaagcgactgtttctgctcctactacaacactggtaggtttgcatagtcag
tgccaacacatttgaatttgaaatctagtcactgtggactcaatagtggcatctaactgatgatcggttgctaaaatttgatttatcatag
tcaaaatgaattatagttggtagc
